# Overview of the Effect of Complementary Medicine on Treating or Mitigating the Risk of Endometriosis

**DOI:** 10.1055/s-0041-1735156

**Published:** 2021-12-21

**Authors:** Firoozeh Mirzaee, Atefeh Ahmadi

**Affiliations:** 1 Nursing Research Center, Department of Midwifery, Razi Faculty of Nursing and Midwifery, Kerman Univeresity of Medical Sciences, Kerman, Iran; 2 Nursing Research Center, Department of Counselling in Midwifery, Razi Faculty of Nursing and Midwifery, Kerman University of Medical Sciences, Kerman, Iran

**Keywords:** complementary medicine, endometriosis, overview, medicina complementar, endometriose, visão geral

## Abstract

**Objective** Endometriosis is a hormone-dependent chronic inflammatory disease with symptoms such as pelvic pain, which affect the physical, emotional, and social health of women in reproductive age. The current overview article aims to explore the effect of complementary medicine on the treatment or in mitigating the risk of endometriosis.

**Methods** This is an overview article done in Iran. Two separate researchers systematically searched 3 databases (Medline, Scopus, and Cochrane Central Register Trials) until September 2020. The methodological quality of each study was assessed using the assessment of multiple systematic reviews (AMSTAR) tool.

**Results** The results of two reviews suggested that physical activity, tobacco smoking, diet, coffee and caffeine intake had no effect on mitigating the risk of endometriosis or improving its treatment, but acupuncture successfully reduced pain and related marker (serum CA-125) levels.

**Conclusion** As endometriosis is an annoying disease with many complications and is hard to diagnose and treat, related studies in complementary medicine can help patients with endometriosis. Based on the relevant literature review, among the complementary medicine available for the treatment or to mitigate the risk of endometriosis, only acupuncture seems to alleviate the pain of endometriosis.

## Resumo

**Objetivo** A endometriose é uma doença inflamatória crônica hormono-dependente com sintomas como dores pélvicas, que afetam a saúde física, emocional e social de mulheres em idade reprodutiva. O presente artigo de visão geral tem como objetivo explorar o efeito da medicina complementar no tratamento ou na mitigação do risco de endometriose.

**Métodos** Trata-se de um artigo de visão geral feito no Irã. Dois pesquisadores separados pesquisaram sistematicamente 3 bancos de dados (Medline, Scopus e Cochrane Central Register Trials) até setembro de 2020. A qualidade metodológica de cada estudo foi avaliada usando a ferramenta avaliação da qualidade dos relatos de revisão sistemática (AMSTAR, na sigla em inglês).

**Resultados** Os resultados de duas revisões sugeriram que atividade física, tabagismo, dieta, consumo de café e cafeína não tiveram efeito na redução do risco de endometriose ou na melhoria do tratamento, mas a acupuntura reduziu com sucesso a dor e os níveis de marcadores relacionados (CA-125 sérico).

**Conclusão** Como a endometriose é uma doença incômoda, com muitas complicações e de difícil diagnóstico e tratamento, estudos relacionados em medicina complementar podem ajudar pacientes com endometriose. Com base na revisão da literatura relevante, entre os medicamentos complementares disponíveis para o tratamento ou risco de endometriose, apenas a acupuntura parece aliviar a dor da endometriose.

## Introduction

Endometriosis is a disease with an estimated incidence of between 6 and 10% in women in reproductive age, ∼ 176 million women worldwide. 1 2 Endometriosis is a hormone-dependent chronic inflammatory disease with symptoms like pelvic pain, which affect the physical, emotional, and social health of women in the reproductive age. 3 4 It has been shown that several pathogeneses including menstrual, genetic, and environmental factors as well as lifestyle play a pivotal role in the development of the disease. 3 4

The symptoms associated with painful endometriosis include dysmenorrhea, nonmenstrual pelvic pain, and painful deep intercourse, which may affect women's quality of life, work, social relationships and sexual function with deleterious implications for the life of these patients and their relationships. 5 6 Despite the high morbidity and healthcare costs associated with this condition, the exact cause of endometriosis remains unknown, although many theories have been developed about the pathophysiological causes of this condition. The risk factors for this condition are largely unidentified.

The existing offered treatments as various pharmacological and surgical therapies 7 are not completely effective. Most of them are prescribed for pain suppression and reduction or reversion of lesions in women suffering from the disease. Some related mechanisms are based on blocking the secretion of estrogen from the ovaries. Oral contraceptives, progestin, danazol, and gonadotropin-releasing hormone agonists (GnRH-a) are used for the treatment of endometriosis, to relieve short-term symptoms. 8 However, these treatments may have considerable side effects, such as menopausal and female climacteric states (2021 ICD-10-CM Diagnosis Code N95.1), such as hot flushes and fatigue.

In addition, danazol can cause androgenic changes, such as acne and weight gain. Previous studies have showed its role in increasing low-density lipoprotein (LDL) cholesterol levels and its conceivable association with ovarian cancer. Gonadotropin-releasing hormone agonists generally decrease estrogen levels more than danazol, and their menopausal related side effects, such as insomnia, hot flushes, low libido, and vaginal dryness are more severe. Low estrogen levels can also lead to serious osteoporosis. The long-term adverse effects of add-back regimes, which use small quantities of progesterone and estrogen, have not yet been completely explored. Patients using progestin treatment suffer more from bloating, acne, spotting, and fluid retention. Progestin may affect the level of high-density lipoproteins (HDL) in the blood, possibly increasing the risk of cardiovascular side effects, such as thrombosis. 7 8

The role of complementary medicine and the development of endometriosis have received growing attention, which is largely due to the physiological and pathological processes related to the disease, including inflammation, estrogen activity, menstrual cycles, organochlorines, and the metabolism of prostaglandin. 9 There are several systematic reviews and meta-analyses on the effect of complementary medicine such as exercise, drinking diet and acupuncture on the endometriosis. In addition, the relationship between tobacco smoking and drinking coffee with endometriosis have been investigated. Therefore, there is a need for simultaneous analysis of several reviews to offer in-depth information to clinicians, policymakers, patients, and researchers. The purpose of the present overview article is to demonstrate the effect of complementary medicine on endometriosis in reproductive age.

## Methods

Six meta-analyses were assessed to identify those that evaluated the effect of complementary medicine on the treatment of endometriosis. Two independent authors did a systematic search of 3 databases (Medline, Scopus, and Cochrane Central Register Trials) until September 2020. The Medical Subject Headings (MESH) keywords searched in English were *exercise* , *tobacco* , *coffee* , *diet* , *complementary medicine* , *acupuncture* , and *endometriosis* . The inclusion criteria were: (1) population (women with endometriosis), (2) intervention (all complementary medicine), (3) results (the effect of complementary medicine on treating or mitigating the risk of endometriosis), and (5) methods (meta-analysis). Duplicate papers were excluded. We also reviewed the references and bibliographies of all studies to find further related studies. The references listed in the reviews, meta-analyses and articles were also manually searched to broaden the scope of the search. The authors who searched the databases and other sources also assessed the quality of the studies and data extraction ( Table 1 ). Before making a final decision, disagreements were settled by consensus. The methodological quality of systematic reviews was also assessed using the 11-item assessment of multiple systematic reviews (AMSTAR) tool developed by Oxman et al. 10 ( Table 2 ). Each item was scored on a 3-point Likert scale (yes, no, and cannot answer). A predesigned form validated by the research team members was utilized to extract the study data, including the type of review, year of publication, first author, study populations, sample size, and main outcomes.

**Table 1:** Characteristics of the six studies included in the present overview

Authors	Year	Type of review	Age range (years old)	Type of intervention	Sample size	Conclusion
Xu et al. 6	2017	Meta- analysis	13–52	Acupuncture, sham acupuncture, Western medicine, traditional chinese medicine	591	Acupuncture reduced pain and had a positive effect on peripheral blood CA-125 levels
Chiaffarino et al. 14	2014	Meta- analysis	15–65	Coffee and caffeine intake	1,407	There was no evidence on the association between coffee/caffeine intake and the risk of endometriosis
Mira et al. 9	2018	Meta- analysis	13–50	Acupuncture, exercise, electrotherapy, and yoga	385	All studies were inconclusive in affirming benefits, but demonstrated a positive trend in the treatment of endometriosis symptoms
Hoorsan et al. 13	2017	Meta- analysis	Reproductive age	Calcium intake, milk, eggs bacon, red meat	72,662	Calcium, milk, eggs, bacon, and red meat intake increase the risk of endometriosis
Ricci et al.	2016	Meta-analysis	Reproductive age	Endometriosis women physical activity	79,55	Physical activity does not reduce the risk of endometriosis
Bravi et al. 11	2014	Meta- analysis	15–56	Tobacco smoking	13,129	No evidence on the association between tobacco smoking and the risk of endometriosis

The study population of all of the studies was composed of women with endometriosis

**Table 2:** Assessment of the methodological quality of systematic reviews according to criteria set by the Center for Evidence-Based Management

AMSTAR items													
References	Year	1 *	2 †	3 ‡	4 §	5 ‖	6 ¶	7 **	8 ††	9 ‡‡	10 §§	11 ‖‖	12 ¶¶
Xu et al. 6	2017	Yes	Yes	No	No	Yes	Yes	Yes	Yes	Yes	Yes	Yes	Yes
Chiaffarino et al. 13	2014	Yes	Yes	Yes	No	Yes	No	Yes	Yes	Yes	Yes	Yes	Yes
Mira et al. 9	2018	Yes	Yes	Yes	No	Yes	Yes	Yes	Yes	Yes	Yes	Yes	Yes
Hoorsan et al. 13	2017	Yes	Yes	Yes	No	Yes	Yes	Yes	Yes	Yes	Yes	Yes	Yes
Ricci et al. 12	2016	Yes	Yes	Yes	No	Yes	Yes	Yes	Yes	Yes	Yes	Yes	Yes
Bravi et al. 11	2014	Yes	Yes	Yes	No	Yes	Yes	Yes	Yes	Yes	Yes	Yes	Yes

Abbreviation: AMSTAR, Assessment of Multiple Systematic Reviews.

* 1: Did the study address a clearly focused question?

† 2: Was a comprehensive literature search conducted using relevant research databases (e.g., ABI/INFORM, Business Source Premier, PsycINFO, Web of Science, etc.)

‡ 3: Is the search systematic and reproducible (e.g., were searched information sources listed, were search terms provided)?

§ 4: Has publication bias been prevented as far as possible (e.g., were attempts made at collecting unpublished data)?

‖ 5: Are the inclusion and exclusion criteria clearly defined (e.g., population, outcomes of interest, study design)?

¶ 6: Was the methodological quality of each study assessed using predetermined quality criteria?

** 7: Are the key features (population, sample size, study design, outcome measures, effect sizes, limitations) of the included studies described?

†† 8: Has the meta-analysis been conducted correctly?

‡‡ 9: Were the results similar from study to study?

§§ 10: Is the effect size practically relevant?

‖‖ 11: How precise is the estimate of the effect? Were confidence intervals given?

¶¶ 12: Can the results be applied to your organization?

## Results

**Fig. 1:**
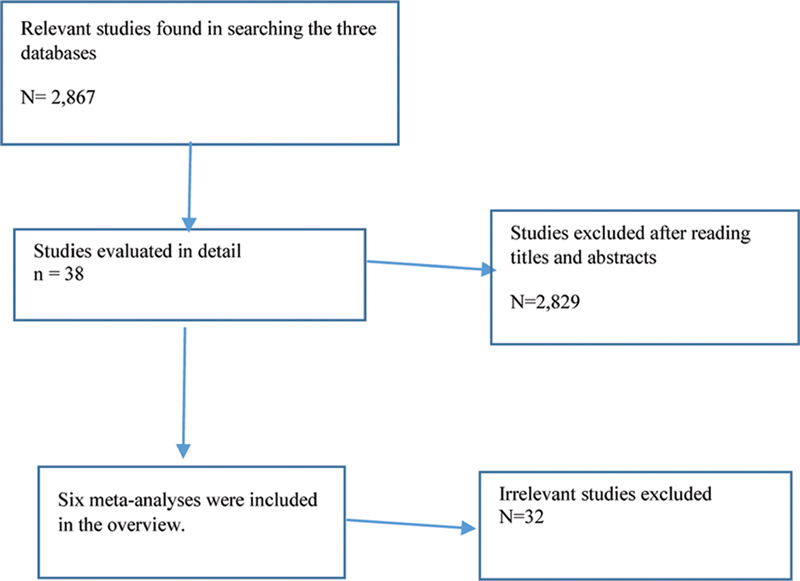
Search strategy of the study.

Fig. 1 shows the process of selecting reviews for the overview. As can be seen, six meta-analyses were included in the overview, as listed in Table 1 . It should be noted that some articles focused on reducing the symptoms of endometriosis and others focused on reducing the risk of endometriosis.

### Physical Activity and Endometriosis

Six case-control and 3 cohort studies included 3,355 cases of recent physical activities, and 4,600 cases were related to past physical activities. The summary odds ratio (OR) for endometriosis according to the physical activity level was calculated by the random-effect model (0.85) [95% confidence interval [CI]: 0.67–1.07] for recent versus lack of physical activities. Compared with lack of recent physical activities, ORs for low and moderate/high physical activities were 1.00 (95%CI: 0.68–1.28) and 0.75 (95%CI: 0.53–1.07), respectively. It is often suggested that physical activity can mitigate the risk of endometriosis, but this hypothesis is not supported by the present meta-analysis. Further research is warranted to lend credit to our findings regarding the benefits of exercise at molecular and endocrine levels, or the effect of related confounding mechanisms such as study design, choice of controls, and physical activity on pain improvement.

### Tobacco Smoking and Endometriosis

In the present paper, summary estimates of the relative risk (RR) are made using the random effect model, and heterogeneity studies are assessed by χ2 test and quantified by I2 statistics. As compared to never-smokers, the summary RR were 0.96 (95% CI 0.86 to 1.08) for ever smokers, 0.95 (95% CI 0.81 to 1.11) for former smokers, 0.92 (95% CI 0.82 to 1.04) for current smokers, 0.87 (95% CI 0.70 to 1.07) for moderate smokers and 0.93 (95% CI 0.69 to 1.26) for heavy smokers. The present meta-analysis provided no evidence for the association between tobacco smoking and the risk of endometriosis. The results are constant considering ever, former, current, moderate and heavy smokers, and across the type of endometriosis and study design. 11

### Diet and Endometriosis

The present systematic review and meta-analysis of studies suggested that the intake of calcium (OR: 0.99) (95%CI: 0.83–1.18), milk (OR: 0.90) (95%CI: 0.65–1.23), eggs (OR: 1.01) (95%CI: 0.81–1.28), bacon (OR: 1.26) (95%CI: 0.60–2.65), and red meat (OR: 1.26) (95%CI: 0.73–2.18), increase the risk of endometriosis. The evidence highlights the prevention impact of dietary components on the risk of endometriosis. Furthermore, more studies are required to explore the role of diet and nutritional elements in the incidence and progression of endometriosis. 13

### Complementary Treatments and Endometriosis

The complementary interventions considered in the present study were acupuncture, exercise, electrotherapy, and yoga. Although, these studies showed effects for the treatment of endometriosis symptoms but all of them yielded inconclusive outcomes. A meta-analysis of acupuncture indicated its benefits in pain reduction compared with placebo ( *p* = 0.007). Several complementary treatments have been used to relieve the symptoms of endometriosis, but only acupuncture has demonstrated a significant improvement in outcomes. Nonetheless, other approaches have also been successful in relieving symptoms. This calls for further efforts to design controlled studies that back up their applicability. 9

### Effect of Coffee and Caffeine Intake on Endometriosis

In the present paper, 8 (6 case-control and 2 cohort) studies, including a total of 1,407 women with endometriosis, were reviewed. The summary relative risks for the intake versus nonintake of caffeine (1.26) (95%:CI: 0.95–1.66) and coffee (1.13) (95%CI: 0.46–2.76) was obtained. The overall guess was 1.18 (95% CI 0.92–1.49). Moreover, the summary relative risks was 1.09 (95%CI: 0.84–1.42) and 1.09 (95%CI: 0.89–1.33) for high and low caffeine intake, respectively, as opposed to nonintake. The present meta-analysis provided no evidence for the association between coffee/caffeine intake and the risk of endometriosis. Therefore, the coffee/caffeine intake, as currently used in diet, does not pose a health risk. 14

### Effect of Acupuncture on Endometriosis

Out of 10 studies reviewed, only 1 pilot study had used a placebo control and blinding. The rest had utilized various controls (medications and herbs), for which blinding was impossible. The sample size was small in all studies, ranging from 8 to 36 patients per arm. The mean difference (MD) in the pain reduction (preminus postinterventional pain level measured on a 0 ± 10-point scale) between the acupuncture and control group was 1.36 (95% confidence intervals [CI] = 1.01-1.72, *p* < 0.0001). Acupuncture had a positive effect on peripheral blood CA-125 levels, compared with the control group (MD = 5.9, 95% CI = 1.56-10.25, *p* = 0.008). Similarly, acupuncture had a positive effect on clinical effective rate, when compared with the control groups (odds ratio = 2.07; 95% CI = 1.24-3.44, *p* = 0.005). Few randomized, blinded clinical trials have addressed the efficacy of acupuncture in treating endometriosis-related pain. However, current studies suggest that acupuncture mitigates pain and serum CA-125 levels, regardless of the type of control intervention used. To corroborate these findings, additional blinded studies with suitable controls and suitable sample sizes are needed. 6

## Discussion

In the present study, six systematic reviews and meta-analyses studying the effects of complementary medicine on endometriosis treatment were reviewed. It is hypothesized that physical activity can increase the levels of sex hormone binding globulin (SHBG), which decreases bioavailable estrogens. 15 16 Steady physical activity also reduces insulin resistance and hyperinsulinemia. Hyperinsulinemia may increase the concentration of estrogens by decreasing the concentration of SHBG and elevating the concentration of insulin-like growth factor-1 (IGF-1), which can stimulate endometrial cell proliferation by dwindling concentrations of insulin-like growth factor binding protein 1 (IGFBP-1). 15 Finally, regular physical activity seems to have protective effects on inflammatory processes and oxidative stress, as it raises systemic levels of anti-inflammatory cytokines. 16 However, the meta-analyses reviewed in the present study do not conclusively support this hypothesis.

The results of the present study provided no evidence for the association between tobacco smoking and the risk of endometriosis. Since endometriosis is an estrogen-dependent disorder, the inverse association between smoking and endometriosis reported in some studies is normally attributed to the antiestrogenic effect of tobacco smoking. 17 Some authors have suggested that estradiol can modify the mediators of immune system molecules or those involved in tissue cell adhesion and invasion. 18

Literature review in this study clarified the need for deeper insights into the impact of dietary components on the endometriosis. What is confirmed about diet is that risk of developing endometriosis drops by greater consumption of fish. In addition, the results of this meta-analysis demonstrated that milk, calcium, and vitamin D have no effect on the risk of developing endometriosis, increase low-fat dairy products, reduce the risk of endometriosis and also increase the levels of 25- hydroxyl-vitamin D3. Also, dairy products reduce the risk of endometriosis. 19 This study indicates the positive effect of these foods on risk mitigation, though none of these results were statistically significant. The results do not support the association between coffee/caffeine intake and the risk of endometriosis. It has been suggested that, in women, caffeine influences the hepatic production of SHBG and elicits subsequent reductions in bioavailable testosterone. 20 Other studies have demonstrated the role of caffeine in inhibiting aromatase, a key enzyme mediating the conversion of androgens to estrogens. 21

Several complementary treatments have been proposed to alleviate the symptoms of endometriosis, but only acupuncture has been able to produce significant positive outcomes. 13 Acupuncture decreases pain and serum CA-125 levels and improves endometriosis by various mechanisms. It seems that this complementary medicine reduces pain by raising pain thresholds in humans. 22 It improves the release of neuro-hormonal factors, including adenosine, γ-aminobutyric acid, opioid peptide, acetylcholine, nitric oxide, noradrenaline, and dopamine. 23 24 In addition, acupuncture suppresses serum estradiol levels. 25 Therefore, it may constrain the growth of ectopic endometrium and relieve pain. The last mechanism fosters the capacity of the immune system to remove malignant cells more actively by strengthening the ability of natural killer (NK) cells to kill cancer cells. 26 More specifically, acupuncture stimulation increases the cytotoxicity of NK cells by promoting crosstalk between the neurotransmitter network and the immune system. Mediated by nitric oxide, β-endorphins, and cytokines, 27 this crosstalk is anchored by opioid and NK cell receptors.

The findings of the present overview should be interpreted with caution because most of the studies has not reported or explained their randomization technique, dropout rate, and attrition rate, no use of intention to treat, blinding method, sequence generation, and sample size estimation method. It is strongly recommended that future research adopts consort criteria to provide high quality results for improving systematic reviews and meta-analyses.

## Conclusion

The present study suggests that, among different complementary medicines like acupuncture, exercise, electrotherapy, and yoga for the treatment of endometriosis, only acupuncture is effective in alleviating endometriosis pain. Furthermore, some types of nutritional elements seem to increase the risk of endometriosis. Physical activity does not reduce the risk of endometrioses, and there is no association between tobacco smoking and the risk of endometriosis.
